# Electroencephalography Based Microstate Functional Connectivity Analysis in Emotional Cognitive Reappraisal Combined with Happy Music

**DOI:** 10.3390/brainsci13040554

**Published:** 2023-03-25

**Authors:** Wangchun Hua, Yingjie Li

**Affiliations:** 1 School of Communication and Information Engineering, Shanghai University, Shanghai 200444, China; 2 College of International Education, Shanghai University, Shanghai 200444, China; 3 School of Life Sciences, Institute of Biomedical Engineering, Shanghai University, Shanghai 200444, China

**Keywords:** EEG, microstate, functional connectivity, happy music, cognitive reappraisal

## Abstract

Currently, research mainly focuses on the effects of happy music on the subjective assessment of cognitive reappraisal, but relevant results of the neural mechanism are lacking. By analysing the functional connectivity of microstates based on electroencephalography (EEG), we investigated the effect of cognitive reappraisal combined with happy music on emotional regulation and the dynamic characteristics of brain functional activities. A total of 52 healthy college students were divided into music group and control group. EEG data and behavioural scores were collected during an experiment of cognitive reappraisal combined with happy music. The dynamic time window of the brain functional network was determined by microstate analysis, and the metrics of functional connectivity, clustering coefficient (Cp) and characteristic path length (Lp), were calculated based on the phase-locked value. The arousal of cognitive reappraisal significantly increased (*p* = 0.005) in music group, but the valence did not change significantly. This suggested that happy music did not affect emotional regulation from the behavioural perspective. Four microstate global templates (A–D) were determined. With happy music, the duration (*p* = 0.043) and Lp (*p* = 0.033) of microstate B increased significantly, indicating that the transfection efficiency of the brain network decreased, reflecting a negative effect on cognitive reappraisal. The duration (*p* = 0.017) of microstate D decreased and of Cp (*p* < 0.001) increased significantly, indicating that the local information-processing ability of the brain network increased. We conclude that happy music can change the characteristics of brain functional networks and have a positive effect on cognitive reappraisal in specific period. The research provides a certain electrophysiological basis for applying happy music to cognitive reappraisal.

## 1. Introduction

Emotions affect both physical and mental health. People who experience negative emotions over a long period of time may have various mental or physical problems or even suffer from diseases. It is medically proven that 76% of diseases are emotionally related [1]. It is difficult to avoid negative emotions in daily life; therefore, effective regulation is crucial. Emotional regulation includes several strategies, among which cognitive reappraisal is considered the most effective. It aims to reduce emotional reactions by changing the understanding of emotional events or perception of personal meaning [2]. Compared to other strategies, cognitive reappraisal has a lasting regulatory effect and strong operability [3]. Currently, cognitive reappraisal is widely used in prevention and intervention for emotional problems. However, it is still unclear how to effectively improve the effect of cognitive reappraisal.

Music is an effective means of eliciting and regulating emotions, and music therapy combined with psychotherapy can significantly improve depressive symptoms [4,5]. Research has shown that happy music can enhance positive emotions [6]. Chin et al. found that people who enjoy listening to music are more likely to engage in cognitive reappraisal to regulate their emotions [7]. Baltazar et al. showed that the combination of music and cognitive reappraisal did not significantly improve the effect of emotion regulation compared with music or emotion regulation alone [8]. Carvalho experimentally demonstrated that, in people who enjoy music, the combination of music and cognitive reappraisal improved emotion regulation [9]. They also found that in cognitive reappraisal, the musical features to which participants focused their attention were not the lyrics of the song, but the feelings and memories triggered by the music, which may increase empathy and facilitate the re-perception of events [9]. Currently, research on music in relation to cognitive reappraisal strategies relies mainly on psychological theories and obtains its results through subjective reports, but lacks evidence of relevant neural mechanisms.

Emotional cognition is a complex process involving multiple brain regions working in a coordinated manner [10,11]. The analysis of brain functional connectivity can globally reveal the cooperative working mechanism between multiple brain regions and help to explore the principles and cognitive laws of the brain in various cognitive tasks [12]. The interaction between brain regions is usually transient and dynamic [13,14]. Functional connectivity analysis ignores the time-varying characteristics of these interactions [15]. Dynamic functional connectivity analysis provides a new perspective for the study of brain neural activity by dividing the states of functional connectivity states into distinct periods [15]. Electroencephalography (EEG) is a non-invasive technique that is highly sensitive to millisecond-level changes in brain functional states [16]. In recent years, the number of EEG studies on dynamic functional connectivity has increased rapidly [17,18]. Fang et al. have demonstrated the dynamic reorganisation of brain functional networks in the process of emotion regulation by analysing dynamic functional connectivity [19]. A microstate is a limited number of different quasi-stable topographies parsed from a multichannel EEG, and considered the shortest cognitive component of spontaneous conscious activity. Microstates can effectively reflect dynamic changes in the global functional state of the brain over time [20].

We used microstates for dynamic functional connectivity analysis to investigate the effects of cognitive reappraisal in combination with happy music from the perspective of electrophysiology. The experimental paradigm was a cognitive reappraisal task with emotional imagery. Participants were divided into music and control groups. Therefore, we hypothesise the following: Happy music may alter the temporal characteristics of the microstates of the cognitive reappraisal process, and subsequently alter the functional connectivity of brain regions. The effect of happy music on cognitive reappraisal was reflected in the behavioural scores. These results may add to the understanding of the neural mechanisms of cognitive reappraisal associated with happy music and contribute to the application of music therapy in emotion regulation.

## 2. Materials and Methods

### 2.1. Participants

In this study, 52 students from Shanghai University were recruited and divided into experimental and control groups. The experimental group consisted of 28 participants, including 13 males and 15 females, with an age of 22.9 ± 1.9 years and an educational level of 16.8 ± 1.9. The control group consisted of 24 participants, including 9 males and 15 females, with age of 22.5 ± 2.3 years and educational level of 17.2 ± 2.1. There were no significant differences in gender, age, or educational level between the two groups. None of the participants had a history of mental or neurological diseases. Neither group has received professional music training. This study was approved by the Shanghai Clinical Research Ethics Committee and complied with the Helsinki Declaration [21]. Prior to the experiment, all participants signed an informed consent form.

### 2.2. Experiment

**Figure 1:**
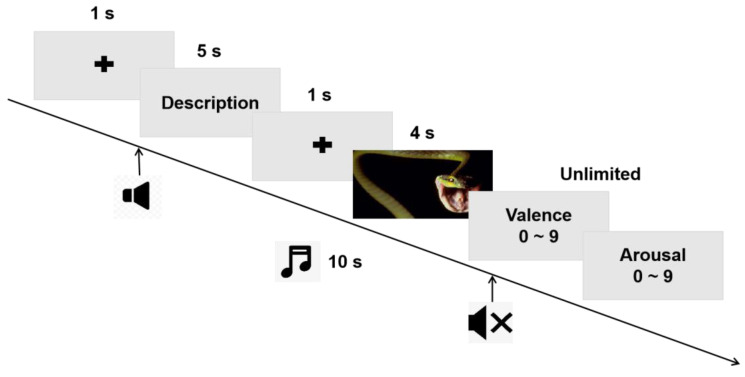
Experimental paradigm of cognitive reappraisal in combination with happy music.

The experimental paradigm was based on the emotional cognitive reappraisal paradigm described in previous study [22,23]. The experimental procedure was compiled using the E-Prime software (version 2.0; Psychology Software Tools, Pittsburgh, PA, USA). Figure 1 shows the procedure. Ninety trials were performed. In each trial, a black fixation cross (1 s) appeared on the screen, followed by a negative or neutral text description (5 s). After the text disappeared, a black cross appear again (1 s), and finally, a negative or neutral image appeared (4 s). After the image disappeared, all participants rated each image according to valence (1 = extremely negative, 9 = highly positive) and arousal (1 = calm, 9 = aroused) according to their own real-life perception of the image in combination with the text description. The 90 trials were divided into 60 negative and 30 neutral pictures. All pictures were selected from the International Affective Picture System (IAPS) [24]. Half of the negative pictures were preceded by negative descriptions (Neg condition) and the other half were preceded by neutral descriptions to achieve cognitive reappraisal (Rea condition). All neutral pictures were preceded by neutral descriptions (Neu condition).

To examine the effect of cognitive reappraisal in combination with happy music, participants were randomly divided into music and control groups. The control group underwent the same experimental procedure as described above. The music group listened to happy music throughout the experiment. A nice, pure music clip (10 s) began to play when the text description appeared and stopped when the image disappeared. All music clips were selected from pure classical Chinese music. An additional 20 students were recruited and scored with a valence of 6.6 ± 0.4 and an arousal of 5.8 ± 1.6.

All participants were instructed to understand the entire experimental procedure before the experiment and to perform the procedure in a closed room without sound interference. The trial order of each participant was randomly disordered, and the music clips of the music group were randomly played.

### 2.3. EEG Recording and Preprocessing

EEG signals were recorded using NeuSen W (Neuracle, Changzhou, Jiangsu, China) with 64 Ag/AgCl electrodes positioned on an elastic cap according to the international extended 10-10 system. The ground electrode was placed between FPz and Fz and the reference electrode was placed between Cz and CPz. Before the experiment, the impedance of each electrode was reduced to less than 5 kΩ by conductive gel. During the experiment, EEG data were recorded at a sampling frequency of 1000 Hz, and a notch filter was used to remove the 50 Hz power frequency.

Preprocessing of the EEG data was performed in MATLAB version R2020b using the EEGLAB toolbox version 14.1.1 [25]. First, the original data were bandpass filtered at 0.1-30 Hz. Channels with excessive noise, drift, or poor connections were interpolated using a spherical interpolation. The data were then divided into epochs from 200 ms pre-stimuli to 5000 ms post-stimuli and corrected for baseline using the pre-stimuli at 200 ms. Next, independent component analysis (ICA) was used to remove artefacts such as eye movements, and bad epochs were removed by visual inspection. Finally, the cleaned data were referenced to the average reference and resampled at 250 Hz. The EEG grand averages for each participant were calculated to obtain ERP under three conditions (Neg, Neu, and Rea) and two groups (music and control).

### 2.4. Microstate Functional Connectivity Analysis

Microstate analysis was performed using the Ragu tool from the EEGLAB microstate plugin (http://www.thomaskoenig.ch/index.php/software/microstates-in-eeglab, accessed on 30 June 2016) in MATLAB version R2020b [26]. Similar procedures can be found in previous descriptions [27,28]. 

First, the global field power (GFP) was calculated at each time point of the ERP curve [29].

(1) $$GFP=\sqrt{\left(\overset{N}{\underset{i=1}{\sum }}\left(V_{i}\left(t\right)-{\bar{V}}_{i}\left(t\right)\right)\right)/N}\;$$

where $V_{i}\left(t\right)$ and $\bar{V_{i}}\left(t\right)$ are the instantaneous and mean potentials across the N electrodes at time t, respectively. GFP represents the instantaneous value of the electric field strength in the brain [20]. Microstates are defined as periods of stable electric field topographies that tend to remain stable near the GFP peak and change rapidly around the local GFP minimum value [20]. Second, the ERP topographies corresponding to each GFP point were submitted to the clustering algorithm, taking into account that ERP are time-locked potentials for event stimuli. The polarity of each topography cannot be ignored. The K-means clustering method was selected as the clustering algorithm rather than the polarity-invariant atomise and agglomerate hierarchical clustering (AAHC) algorithm [30] because ERP components have positive and negative polarities. To determine the optimal number of microstate prototypes, a cross-validation criterion was applied to all participants. The procedure was performed 50 times, varying the number of clusters from 3 to 10. Each time, the participants were randomly split into 50% training and 50% testing datasets. The global exact variance (GEV) was used to determine the optimal number of microstates [31]. In the third step, all individual microstate classes of each participant were averaged to obtain the global mean template according to the permutation algorithm [32]. The individual microstate classes were then sorted according to the global mean template. Finally, the global mean template was fitted to each time point on the GFP curve to determine the dynamic time range for each microstate. The ERP topography of each point was assigned to a template class by determining the maximum spatial correlation coefficient between the topography and global mean template. The dynamic time window of each microstate under different conditions was determined and the duration of each microstate was analysed.

The phase-locking value (PLV) between signals in different channels was calculated to construct a time-varying functional connectivity network. The PLV describes the phase synchronisation of two EEG signals and ranges from 0 to 1 [33]. It is defined as

(2) $$PLV\left(t\right)=\frac{1}{N}\left|\overset{N}{\underset{k=1}{\sum }}e^{i\left(\varphi_{\left(t,k\right)}-\psi_{\left(t,k\right)}\right)}\right|$$

where N represents the number of trials, $\varphi_{\left(t,\;k\right)}$ and $\psi_{\left(t,\;k\right)}$ are the instantaneous phase of the kth EEG epoch of channel φ and ψ at time point t, obtained by the Hilbert transform of the signal. The average PLV of each microstate was calculated by averaging the PLVs at the time points with the same microstate label. The clustering coefficient (Cp) and the characteristic path length (Lp) of the functional connectivity network based on the PLV were calculated using the GRETNA toolbox [34]. Cp is the fraction of triangles around a node, and the average clustering coefficient of a network is the mean Cp value of all nodes in the network. A larger mean Cp value reflects higher local information processing in a network. Lp is the average shortest path length between all pairs of nodes in the network and reflects the global integration of the network. A shorter Lp value indicates higher information transformation efficiency in the brain network [27].

### 2.5. Statistics

All statistical analyses were performed using IBM SPSS version 22. The significance level was set at *p* < 0.05. To test the effects of cognitive reappraisal in combination with happy music, a two-way repeated-measures ANOVA (rmANOVA, Condition*Group) was conducted. Condition effects included Neg, Neu, and Rea, whereas the group effects included control and music. Greenhouse–Geisser correction was performed when the factors analysed did not meet the Mauchly test for sphericity. Post hoc multiple comparisons with Bonferroni correction were performed to determine group differences in each condition. 

## 3. Results

### 3.1. Behavioural Results

**Figure 2:**
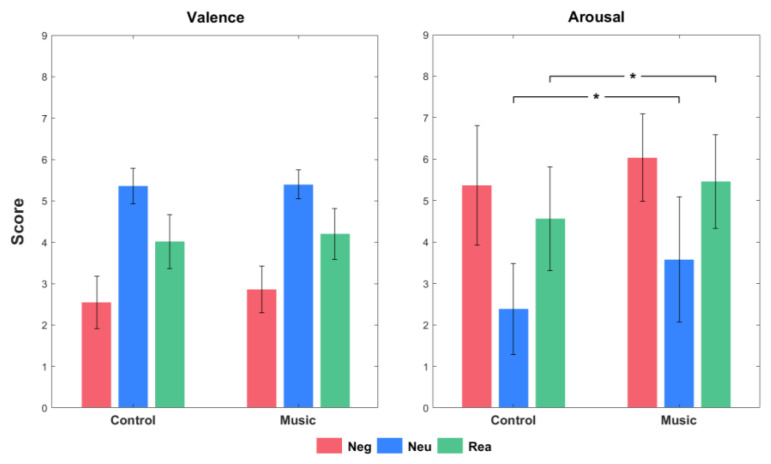
Behavioural results of the different conditions in the control and music group (annotations * denotes the significant interaction effect, *p* < 0.05).

Valence mainly reflects emotional regulation, while arousal mainly reflects emotional perception. The valence and arousal scores for the different conditions in the control and music groups are shown in Figure 2. For valence, a main effect of condition (F(2, 100) = 379.250, *p* < 0.001) was found. Rea had higher scores than Neg (*p* < 0.001), and both Neg and Rea had lower scores than Neu (*p* < 0.001). The valence of Rea significantly increased compared to that of Neg, indicating that both groups achieved cognitive reappraisal. The main effect of Group (F(1, 50) = 5.850, *p* = 0.019) showed that the scores for valence were higher in the music group than those in the control group, reflecting that happy music could regulate emotions. However, no significant interaction effect of valence was found. For arousal, a main effect of condition (F(1.391, 70) = 259.113, *p* < 0.001) was found. Rea had lower scores than Neg (*p* < 0.001) and both Neg and Rea had higher scores than Neu (*p* < 0.001). The main effect of Group (F(1, 50) = 9.312, *p* = 0.004) showed that the arousal scores were higher in the music group than those in the control group. A significant interaction effect between condition and group was also found (F(1.391, 70) = 3.944, *p* = 0.038). Multiple comparisons revealed that the arousal scores of both Neu and Rea in the music group were higher than those of the control group (*p* < 0.001 and *p* = 0.005, respectively; Figure 2). It indicated happy music only enhanced emotional perception during the processing of cognitive reappraisal.

### 3.2. Microstate Results

**Figure 3:**
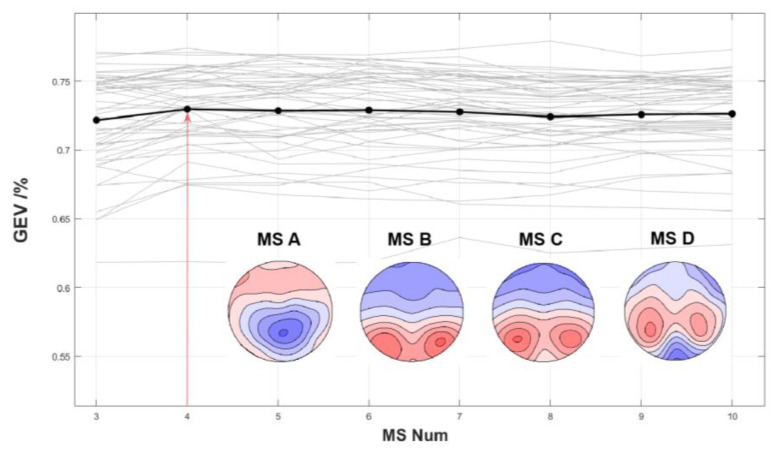
Topographies of the five global microstates across conditions and groups.

As shown in Figure 3, four microstates, labelled MS A-D, were determined according to the cross-validation criteria. Based on the voltage distribution in the microstate topography, MS A has a central-parietal orientation. MS B and C had bilateral occipital orientations. MS B was more inclined toward the right occipital lobe, whereas MS C was more inclined toward the left occipital lobe. Patients with MS D had bilateral parietal orientations.

**Figure 4:**
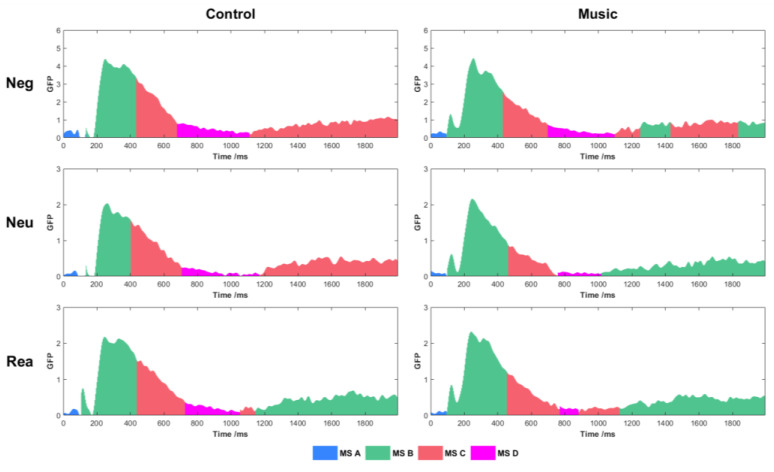
Dynamic time windows of microstates under the GFP curve.

Figure 4 shows the average GFP curve, with different colours indicating the dynamic time windows of each global mean microstate under the curve. The results of the rmANOVA of microstate duration are given in Table 1. A significant main effect of condition was observed for MS A (F(2, 100) = 3.803, *p* = 0.026). The duration for Rea was shorter than that for Neu (*p* = 0.047). No significant group effects or interaction effects were observed. A significant main effect of condition was observed for MS B (F(2, 100) = 3.393, *p* = 0.038). The duration of Rea was longer than that of Neg (*p* = 0.033). A significant main effect of group (F(1, 50) = 3.236, *p* = 0.043) showed that the duration of the music group was longer than that of the control group. No significant interaction effects were observed. A significant main effect of the condition was observed for MS C (F(2, 100) = 3.371, *p* = 0.038). The duration of Rea was shorter than that of Neg (*p* = 0.028). No significant group effects or interaction effects were observed. A significant main effect of condition was observed for MS D (F(2, 100) = 4.830, *p* = 0.010). The duration of Rea was shorter than that of Neg (*p* = 0.021) and Neu (*p* = 0.029). A significant interaction effect (F(2, 100) = 3.589, *p* = 0.033) showed that in the music group, the duration of Rea was shorter than that of Neg (*p* = 0.017) and Neu (*p* = 0.044). No significant group effects were observed.

The duration of both MS B, C, and D were affected by cognitive reappraisal. For MS B, the longer duration in Rea showed more interactions in the functional network. MS C and D showed opposite results to that of MS B, which reduced interactions in the functional network. Happy music had an effect on the duration of MS B and D. Additionally, only MS D showed the effect of happy music on cognitive reappraisal, which led to fewer interactions in the functional network in Rea.

### 3.3. Functional Conectivity Results

**Figure 5:**
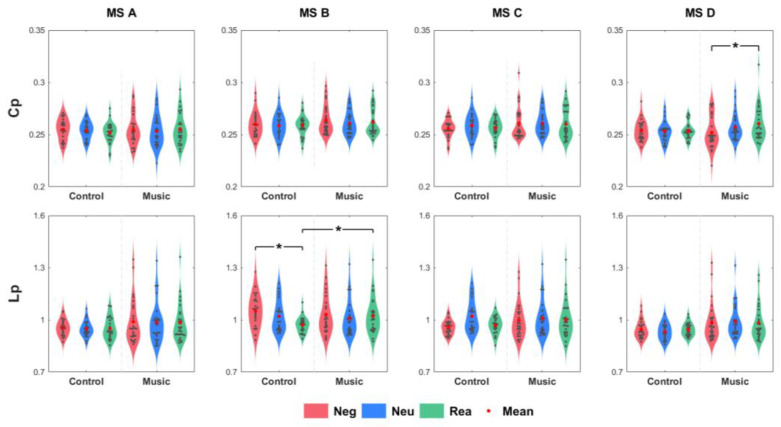
Cp and Lp statistical data of microstates (annotations * denote the significant interaction effect, *p <* 0.05).

Figure 5 shows the values of Cp and Lp for each microstate under different conditions. The results of the rmANOVA are given in Table 2. For MS A, no significant effects of Cp or Lp were observed. For MS B, Lp had a significant main effect on the condition (F(2, 100) = 15.061, *p* < 0.001). The Lp of Neg was greater than that of the Neu (*p* = 0.004) and Rea (*p* < 0.001) groups. Lp also showed a significant interaction effect (F(2, 100) = 11.039, *p* < 0.001). In the control group, the Lp of Neg was greater than that of Neu (*p* = 0.013) and Rea (*p* < 0.001), and the Lp of Neu was greater than that of Rea (*p* < 0.001). Compared with the control group, the Lp of the music group was greater in Rea (*p* = 0.033). In MS C, Lp had a significant main effect on condition (F(2, 100) = 3.759, *p* = 0.027). The Lp of Neg was lower than that of Rea (*p* = 0.048). For MS D, Cp had a significant main effect on the condition (F(1.731, 86.526) = 8.841, *p* = 0.001). The Cp of Neg was significantly lower than that of Rea (*p* = 0.002). Cp also showed a significant interaction effect (F(1.731, 86.526) = 8.586, *p* = 0.001). In the music group, the Cp of Neg was lower than that of Neu (*p* = 0.032) and Rea (*p* < 0.001), and the Cp of Neu was lower than that of Neg (*p* = 0.001). In addition, a significant main effect of group (F(1, 50) = 4.067, *p* = 0.049) showed that the Lp was greater in the music group than that in the control group.

The Lp of MS B and C were affected by cognitive reappraisal. For MS B, shorter Lp in Rea showed an improvement of information transformation efficiency of the functional network. MS C showed opposite results to that of MS B, which reduced the information transformation efficiency. Both MS B and C results showed that happy music led to a reduction in the efficiency of information transformation. Moreover, Cp results showed that cognitive reappraisal was affected by happy music in MS D.

## 4. Discussion

### 4.1. Behavioural Effect of Happy Music on Cognitive Reappraisal 

Both happy music and cognitive reappraisal strategies regulate negative emotions [2,6]. Our behavioural results did not find significant difference in valence of cognitive reappraisal due to happy music. The increase in arousal could be due to a stronger emotional perception by the happy music and did not reflect a positive effect on cognitive reappraisal. Participants with negative emotions could not perceive the emotional feelings of happy music [35], which could explain why no significant difference in arousal was found in the Neg condition between the two groups. Baltazar et al. found that the combination of music and cognitive reappraisal did not significantly improve emotion regulation [8]. Although happy music can enhance positive emotions, it may be that when listening to happy music and cognitive reappraisal tasks simultaneously, there is a visually dominant effect in the process of multisensory fusion [36], so that the music is not able to effectively evoke individuals’ memories of beautiful scenes, and thus does not have a significant effect on the effect of emotional regulation. Another explanation is that external emotions influence information processing only when participants believe that the emotions are relevant, and that external emotions can influence information processing [37]. When participants perform cognitive reappraisal tasks, they may consider the background music irrelevant; therefore, they will not incorporate the influence of happy music into the emotional regulation process. Carvalho found that for people who enjoy music, a combination of music and cognitive reappraisal can improve emotion regulation [9]. Most importantly, the effect of happy music on cognitive reappraisal was influenced by individual differences. For certain populations, happy music may play a positive role in the emotional regulation of cognitive reappraisal.

### 4.2. Altered Functional Connectivity during Different Microstates

Studies have shown that music can change functional connectivity [38,39]. Based on the analysis of functional connectivity of microstates, we found that happy music affects the characteristics of the brain functional network during the process of cognitive reappraisal. The clustering results showed four microstate templates. The results of MS A did not show any significant effect of happy music. It indicated that both the music and control groups had the same brain processing of emotional responses in the early phase of event stimulation. For MS B, cognitive reappraisal led to prolongation of duration and improvement of information transformation efficiency of the functional network. However, happy music reduced the information transformation efficiency. At the same time, the duration increased significantly in the music group compared with the control group, which may indicate that the information exchange between brain regions requires more time to compensate for the reduction in efficiency. The results of MS C showed that both groups experienced consistent cognitive reappraisal effects during the period, which decreased the duration and efficiency of information transformation. For MS D, happy music improves the local information-processing ability of the functional network. Moreover, the shortened duration of information interaction may also be related to the improved information-processing ability.

Kalisch proposed an “execution-maintaining model” of cognitive reappraisal. The reappraisal strategy is selected and initially implemented in the early stage, and successful maintenance of the strategy occurs in the middle-to-late stage [40]. MS A appeared within 150 ms of stimulation, corresponding to early ERP components, such as P100/N100. The P100 is sensitive to visual stimuli, while the N100 is related to processing responses to attention and emotional stimuli [41]. This suggests that early attentional mobilisation and visual processing of emotional stimuli during the MS A period had not yet completed the selection and implementation of cognitive reappraisal strategies. MS B and C appeared alternately approximately 150 ms after stimulation, reflecting the successful implementation of cognitive reappraisal and the differential effects in the maintenance process. The duration and transformation efficiency of MS B increased, whereas the duration and transformation efficiency of MS D decreased. Britz et al. [42] found that four classic microstate prototypes correspond to the voice processing, visual, saliency default, and attentional networks. In this study, the topographic distribution of MS B and C showed a bilateral occipital orientation. We interpreted MS B and C as the synthesis of voice processing and the visual network; MS B corresponds to the visual network (more right occipital orientation), and MS C corresponds to a voice processing network (more left occipital orientation). Our results suggest that during the maintenance stage of the cognitive reappraisal strategy, the visual network is more activated and the voice processing network is inhibited. The duration and transformation efficiency of MS B were reduced by the happy music, suggesting that cognitive reappraisal was negatively affected. The cognitive interference hypothesis states that performing emotional regulation tasks with background music distracts individuals’ attention, resulting in poor coordination of brain networks [43]. It is important to note that MS D was a special period that occurred at around 1000 ms. The control group failed to maintain the effect of cognitive reappraisal, while happy music improved the ability of local information processing and shortened the time of information interaction. This suggests that cognitive reappraisal was positively affected by happy music during the MS D period.

## 5. Conclusions

The effect of happy music in combination with cognitive reappraisal is not clear. To the best of our knowledge, the study is the first to investigate the effect of happy music on the dynamic functional connectivity characteristics of cognitive reappraisal through electrophysiological methods. Our results showed that happy music did not significantly alter the effect of emotional regulation from the behavioural perspective, but enhanced perception of emotion. Using functional connectivity analysis of ERP microstates, we successfully found that the dynamic characteristics of brain functional network affected by happy music during the processing of cognitive reappraisal. Different microstate periods showed various effects, with happy music reducing the global transmission capability in MS B and increasing the ability to process local information of the functional network in MS D. Happy music can have a positive effect on brain functional network during the processing of cognitive reappraisal, providing a certain electrophysiological basis for applying happy music to cognitive reappraisal. In the future, happy music could be supplemented at specific periods in the processing of cognitive reappraisal to further expand its positive effect on brain functional network, in order to improve the effect of emotional regulation.

In the study, we did not focus on the individual differences of participants regarding the music. A specific population with professional music training could be recruited to investigate whether the positive effect of happy music on brain functional networks can be improved. Moreover, the effects of applying happy music in other research works are also worth exploring.

## Figures and Tables

**Table 1:** The significant rmANOVA results of microstate duration.

MS	rmANOVA		Multi-Comparison	
A	Condition	F(2, 100) = 3.803, *p* = 0.026	Rea < Neu, *p* = 0.047	
B	Condition	F(2, 100) = 3.393, *p* = 0.038	Rea > Neg, *p* = 0.033	
	Group	F(1, 50) = 3.236, *p* = 0.043	Music > Control	
C	Condition	F(2, 100) = 3.371, *p* = 0.038	Rea < Neg, *p* = 0.028	
D	Condition	F(2, 100) = 4.830, *p* = 0.010	Rea < Neg, *p* = 0.021 Rea < Neu, *p* = 0.029	
	Condition*Group	F(2, 100) = 3.589, *p* = 0.033	Music	Rea < Neg, *p* = 0.017 Rea < Neu, *p* = 0.044

**Table 2:** The significant rmANOVA results of Cp and Lp.

MS	Metric	rmANOVA		Multi-Comparison	
B	Lp	Condition	F(2, 100) = 15.061, *p* < 0.001	Rea < Neg, *p* < 0.001 Neu < Neg, *p* = 0.004	
		Condition*Group	F(2, 100) = 11.039, *p* < 0.001	Control	Rea < Neg, *p* < 0.001 Neu < Neg, *p* = 0.013 Rea < Neu, *p* < 0.001
				Rea	Music > Control, *p* = 0.033
C	Lp	Condition	F(2, 100) = 3.759, *p* = 0.027	Rea > Neg, *p* = 0.048	
D	Cp	Condition	F(1.731, 86.526) = 8.841, *p* = 0.001	Rea > Neg, *p* = 0.002	
		Condition*Group	F(1.731, 86.526) = 8.586, *p* = 0.001	Music	Rea > Neg, *p* < 0.001 Neu > Neg, *p* = 0.032 Rea > Neu, *p* = 0.001
	Lp	Group	F(1, 50) = 4.067, *p* = 0.049	Music > Control	

Note: no significant effects of rmANOVA (*p* > 0.05) in MS A.

## Data Availability

Not available.
